# Depletion of the actin bundling protein SM22/transgelin increases actin dynamics and enhances the tumourigenic phenotypes of cells

**DOI:** 10.1186/1471-2121-13-1

**Published:** 2012-01-18

**Authors:** Oliver Thompson, Jeelan S Moghraby, Kathryn R Ayscough, Steve J Winder

**Affiliations:** 1 Department of Biomedical Science, University of Sheffield, Firth Court, Western Bank, Sheffield, S10 2TN, UK; 2 Department of Molecular Biology and Biotechnology, University of Sheffield, Firth Court, Western Bank, Sheffield, S10 2TN, UK; 3 College of Medicine, King AbdulAziz Medical City, National Guard Health Affairs, Riyadh, KSA

**Keywords:** podosomes, invasion, cell motility, reactive oxygen species, tumour suppressor

## Abstract

**Background:**

SM22 has long been studied as an actin-associated protein. Interestingly, levels of SM22 are often reduced in tumour cell lines, while they are increased during senescence possibly indicating a role for SM22 in cell fate decisions via its interaction with actin. In this study we aimed to determine whether reducing levels of SM22 could actively contribute to a tumourigenic phenotype.

**Results:**

We demonstrate that in REF52 fibroblasts, decreased levels of SM22 disrupt normal actin organization leading to changes in the motile behaviour of cells. Interestingly, SM22 depletion also led to an increase in the capacity of cells to spontaneously form podosomes with a concomitant increase in the ability to invade Matrigel. In PC3 prostate epithelial cancer cells by contrast, where SM22 is undetectable, re-expression of SM22 reduced the ability to invade Matrigel. Furthermore SM22 depleted cells also had reduced levels of reactive oxygen species when under serum starvation stress.

**Conclusions:**

These findings suggest that depletion of SM22 could contribute to tumourigenic properties of cells. Reduction in SM22 levels would tend to promote cell survival when cells are under stress, such as in a hypoxic tumour environment, and may also contribute to increases in actin dynamics that favour metastatic potential.

## Background

Smooth muscle protein of 22kDa (SM22α) was one of three protein isoforms (α,β,γ) first purified from chicken gizzard muscle but with no known function [1]. Several years later SM22 was rediscovered and named transgelin due to its apparent ability to induce gelation of actin filaments *in vitro *[2] however subsequent analysis revealed that SM22 and transgelin were one and the same protein [3,4]. These and subsequent analyses have identified a family of related proteins variously known as mp20, NP22, NP25, p27, SM22α, SM22β, transgelin and WS3-10 (see [5] for brief review). Following the sequencing of various vertebrate genomes, it is now recognized that all these proteins arise from just three genes named TAGLN1-3, with SM22α, transgelin and WS3-10 being independently discovered, but identical products of the TAGLN1 gene, SM22β the product of TAGLN2 and the neuronally expressed NP22 and NP25 the product of TAGLN3. SM22 is a member of the calponin family of proteins [6] all of which comprise an amino-terminal calponin homology (CH) domain and from one (SM22) to three (calponin) short motifs know as calponin or CLIK23 repeats [7]. All family members are actin binding and under some circumstances actin bundling proteins. Despite the presence of a CH domain, actin binding is not mediated by the CH domain [8] but through sequences between the CH domain and the first calponin repeat and within the calponin repeats themselves (reviewed in [9]). SM22 orthologues in invertebrates have also been described including mp20 in *Drosophila *and Scp1p in *S. cerevisiae *[10-12]. Unlike the situation in vertebrates with three TAGLN genes, in *S. cerevisiae *Scp1p is the sole SM22/transgelin representative. Studies on Scp1 have shown that deletion of *scp1 *enhances cell longevity through an increase in F-actin turnover and a drop in levels of cell reactive oxygen species. Conversely, increased levels of Scp1 led to decreased actin dynamics, an increase in cellular levels of reactive oxygen species and increased cell death [13]. In addition, it has been observed that SM22 levels are elevated in senescent mammalian cells suggesting that the role of the interaction characterized between Scp1 and actin in yeast may play a similar role in higher eukaryotes [14-16]. We therefore investigated the relationship between SM22/transgelin and the organization of the actin cytoskeleton, cell migration and response to stress in fibroblast and prostate cancer cell lines.

## Results

### Expression of SM22/transgelin family products in cell lines

**Figure 1:**
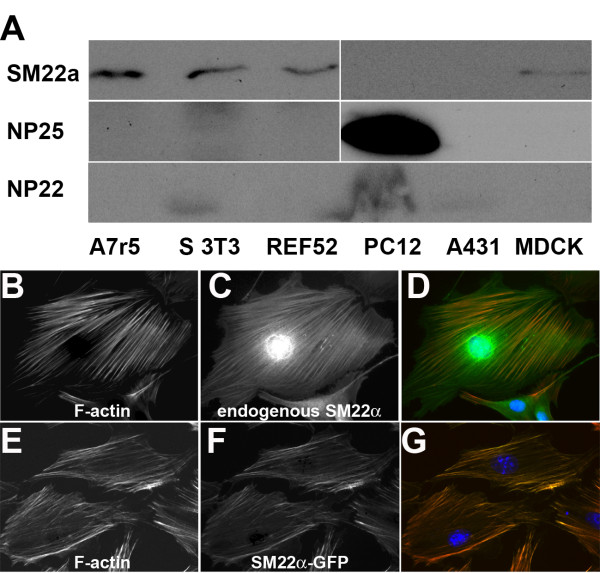
**Expression and localization of transgelin family proteins in REF52 cells**. A, western blot analysis of SM22, NP25 and NP22 levels in various cell lines as indicated (S3T3 = Swiss 3T3). REF52 cells were chosen for further analysis due to their amenable phenotype and the expression of only SM22α. B-G, REF52 cells stained with Alexa 488 phalloidin (red in merge) to reveal F-actin containing structures (B, E), and stained for endogenous SM22α (C), or expressing SM22α-GFP (F) green in merges. Merged images (D, G) all show strong co-localization between endogenous and exogenous SM22 proteins and F-actin stress fibres. Nucleus counterstained with DAPI (blue).

Most vertebrate genomes contain 3 TAGLN genes and many tissues and cells express more than one gene. In order to examine the function of single TAGLN gene products and to increase the chances of being able to deplete all TAGLN gene products in a given cell, we made an initial screen of cell lines from different tissue sources in order to determine what complement of TAGLN gene products were expressed. After analyzing cell lines of fibroblast, muscle, neuronal and epithelial origin, we selected the rat embryo fibroblast cell line REF52 as suitable for further study. This was not only due to its flattened morphology and well organized actin stress fibre morphology that would aid morphological characterization of effects on the actin cytoskeleton, but also based on western blotting showing the presence of only one of the TAGLN gene products namely SM22α (Figure 1A). In keeping with previous reports that SM22 is an F-actin binding/bundling protein, both the endogenous SM22α, revealed by specific antibody staining, and an exogenous SM22α-GFP construct localized strongly with the prominent F-actin containing stress fibres in these cells (Figure 1B-G).

### Depleting SM22 expression levels alters actin organization

**Figure 2:**
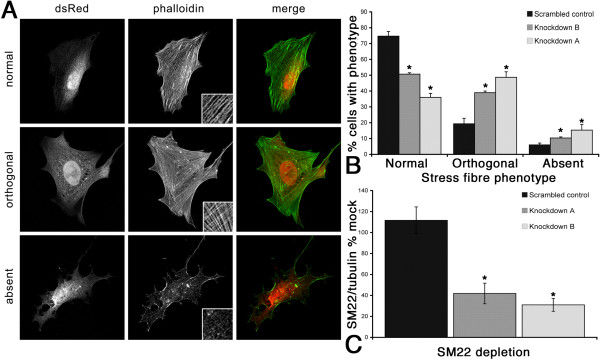
**SM22α depletion alters stress fibre organization**. Examples of the 'normal' 'orthogonal' and 'absent' stress fibre phenotypes in response to depletion of SM22 in REF52 cells. dsRed marks the cells expressing the RNAi, and actin stress fibres are visualized with FITC phalloidin, insets represent examples of the actin phenotypes described (A). Quantification of the stress fibre phenotype shows that depletion of SM22α in two independent cell lines leads to a reduction in normal stress fibres and an increase in the cells falling into the 'orthogonal' or 'absent' categories compared to cells expressing a scrambled RNAi sequence (B). C, quantification of the extent of SM22α depletion by ratio to tubulin content by western blotting in REF52 cells as a % of a mock transfected control (dsRed empty vector). The scrambled RNAi did not deplete SM22α compared to control whereas two independent dsRed SM22α RNAi lines of cells, knockdown A and knockdown B, showed a significant reduction of SM22α levels to 40 and 30% respectively. Mean ± SEM of 3 independent experiments; * p < 0.05 as compared to scrambled control in all cases in B and C.

In order to further dissect the role of SM22, we used siRNA-mediated SM22 depleted REF52 cells to investigate the contribution of SM22 to the actin morphology. Depletion of SM22 to between 20% and 40% of normal levels resulted in a dramatic and reproducible change in actin stress fibre morphology (Figure 2 and Additional file 1, Figure S1). In clones of REF52 cells depleted for SM22, using either REF52 stably expressing siRNA constructs directed against SM22 (Additional file 1, Figure S1) or transient RNAi expression driven from a pSiren-DNR-dsRed plasmid (Figure 2), there was a reproducible and qualitatively similar reduction in SM22 with concomitant loss of actin stress fibre organization. In greater than 70% of wild-type cells actin was organized in longer relatively parallel arrays of fibres, the typical morphology for this cell type (Figure 2A, B). However, in two independent clones (Knockdown A and B; Figure 2) of SM22 depleted cells there was a significant increase in the proportion of cells observed with shorter and orthogonal arrays of actin fibres. These cells also had a marked increase in the proportion of cells with no apparent stress fibres. See insets in Figure 2A for detail of the phenotypes. These observations demonstrate that SM22 has a significant role in the organization of the actin cytoskeleton, and that reduction in SM22 levels leads to a less bundled, less well organized and potentially more dynamic actin cytoskeleton.

### Migration, chemotaxis and invasion in SM22 depleted cells

**Figure 3:**
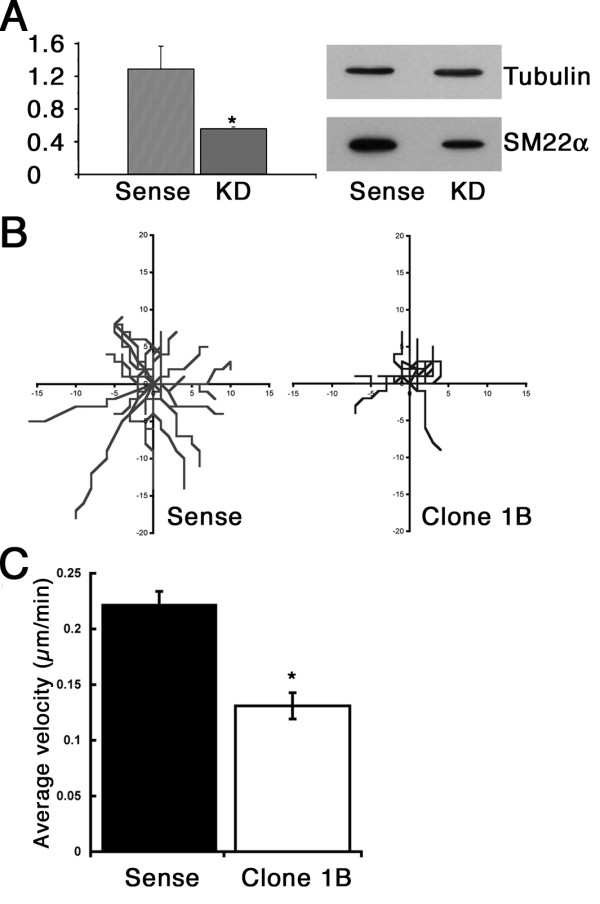
**Effect of SM22α depletion on cell motility**. Stable depletion of SM22α by expressing shRNA achieved 57% knockdown in REF52 cell Clone 1B, compared to cells expressing a control sense RNA hairpin (A). Cell migration was inhibited in SM22 knockdown cells, single cell trajectories were reduced in Clone 1B compared to sense control (C), which was matched by a significant reduction in average velocity (D). Data shown are mean ± SEM in A, C. A n = 3, p < 0.05 compared to Sense, C n = 20, p < 0.02 compared to Sense.

Given the importance of the actin cytoskeleton in cell motility, and that SM22 levels appear altered in tumour cells that often have altered migratory properties, we next investigated the effect of reducing SM22 levels on various in vitro measures of cell migration, chemotaxis and invasion. For improved consistency of cellular response in these experiments REF52 cells stably expressing shRNA were used with a resulting 50% depletion of SM22 compared to sense control (Figure 3A). Single cell tracking of individual SM22 depleted cells revealed that the SM22 depleted cells showed impaired movement with considerably reduced trajectories compared to the sense control. This data was then used to calculate cellular velocities, and as expected revealed that both SM22 depleted clones had significantly reduced average velocity compared to that of the sense control (Figure 3D). Similar effects on cell migration have been noted for vascular smooth muscle cells depleted for SM22 [17].

**Figure 4:**
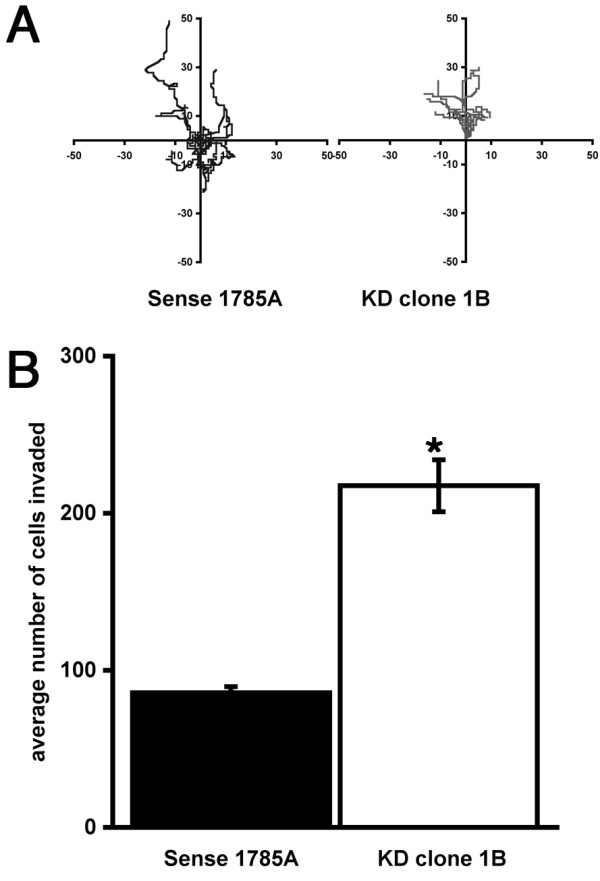
**SM22α depletion results in increased cell migration/invasion**. Despite a reduction in cell velocity REF52 cells depleted for SM22α were able to respond to a serum gradient in Dunn chemotaxis chamber assays (A). However, in invasion assays in Matrigel-coated Boyden chamber assays SM22 depleted cells exhibited a significantly enhanced capacity to invade (B; mean ± SEM 3 independent experiments, * p < 0.01 compared to sense control).

We also analyzed whether there was a change in chemotactic response in SM22 depleted cells. Loss of actin organization can affect not only the mechanics of cell migration, but also cell polarity, which is required for productive directional migration. Cells exposed to a serum gradient in a Dunn chamber assay showed no significant defect in their chemotactic response, indeed Clone 1B appeared to have a higher persistence ratio than wildtype cells (Figure 4A) but nonetheless still migrated a shorter distance. However, motility assays carried out on rigid two-dimensional substrates do not always reflect the motility and invasion of cells in three dimensions which would be more analogous to the *in vivo *situation. We therefore exposed SM22 depleted cells to a chemotactic gradient in Matrigel coated Boyden chambers. Interestingly, in this environment the SM22 depleted cells were able to invade and migrate through the Matrigel much more readily than the control cells (Figure 4B). Therefore despite the inability to migrate and the relative lack of responsiveness to chemotactic stimuli on hard two-dimensional substrates, SM22 depleted cells appear to be much more effective at migrating through soft substrates in response to a similar chemotactic stimulus.

### Podosomes form spontaneously in SM22 depleted cells

**Figure 5:**
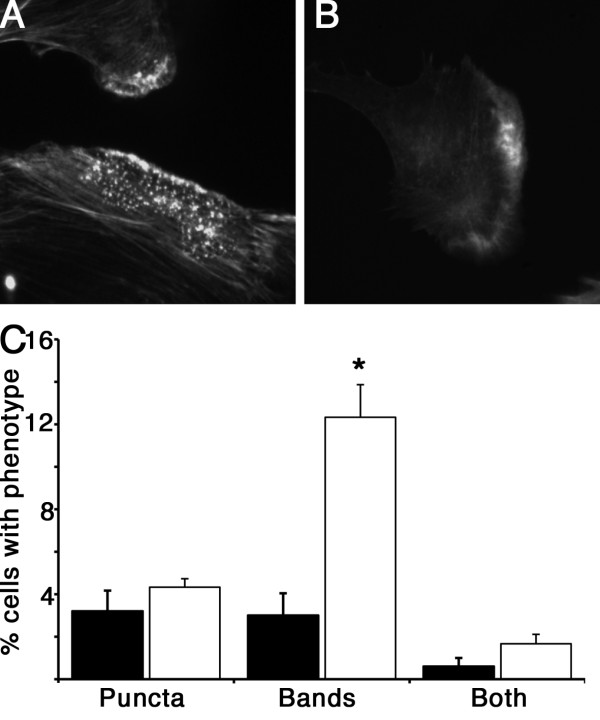
**REF52 cells depleted for SM22 have an altered actin morphology**. SM22 depleted cell show an increase in dense actin puncta reminiscent of podosomes (A), and cells with peripheral actin bands (B), or both, but only the increase in peripheral actin bands was statistically significant. Quantitative data shown in C, represent at least 100 cells counted in each of 5 independent experiments, mean ± SEM, * p < 0.05 compared to control shRNA. Black bars, control shRNA, white bars knockdown clone 1B.

Podosomes, and the related structures invadopodia, are typically found in macrophages, osteoclasts and tumour cells and are not usually seen in other mesenchymal cells unless transformed for example with active Src [18]. Smooth muscle cells and myoblasts however, have been shown to form podosomes under certain non-transformed conditions, including following phorbol ester or TGF-β stimulation [19-22]. On examining the actin staining pattern of SM22 depleted cells in more detail, we noted in addition to the change from the prominent stress fibre phenotype seen in wild type and control REF52 cells, to the less organized actin morphology of SM22 depleted cells (Figure 2), peripheral bands of diffuse F-actin staining and dense puncta of F-actin were now observed with higher frequency (Figure 5A, B). These F-actin puncta and diffuse clouds of F-actin are reminiscent of podosomes and F-actin rosettes seen in other migratory and invasive cells. Quantification of these additional actin phenotypes demonstrated that there was a increase in both lamellipodial rosettes and/or spontaneous podosome formation in cells depleted for SM22 (Figure 5C) although only the increase in peripheral actin bands was significant. These findings demonstrate a further change in cellular actin structures in response to SM22 depletion. The relatively static stress fibre phenotype with associated focal adhesions changes to a more dynamic F-actin based adhesion system is seen by the appearance of more podosomes and rosettes in SM22 depleted cells.

### Depleting SM22 expression levels alters levels of reactive oxygen species

**Figure 6:**
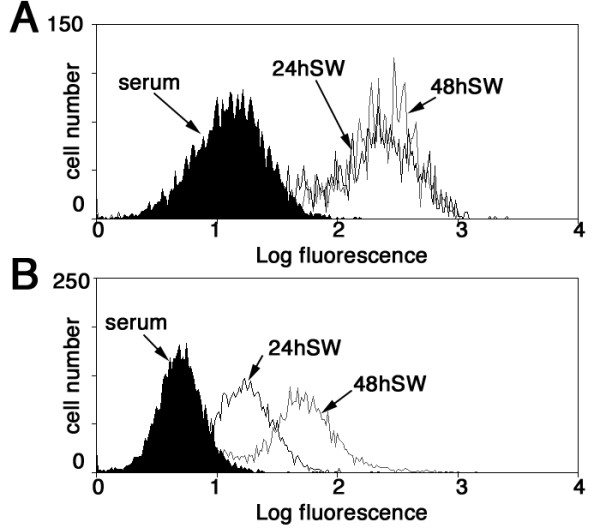
**SM22 depletion reduces stress induced ROS production**. ROS levels were measured in control REF52 cells (A) or SM22-depleted REF52-depleted cells (B) in normal serum conditions or following serum withdrawal for 24 or 48 h. In control REF52 cells both 24 h or 48 h serum withdrawal resulted in a 15-fold increase in ROS levels. REF52 cells depleted for SM22 (B) however, had 66% lower intrinsic ROS levels, and a considerably reduced ROS response to serum withdrawal for 24 h or 48 h (4-fold and 11-fold increase over untreated respectively). Control cell population is shaded in black, 24 and 48 serum withdrawal populations (24SW and 48SW) are indicated by black or grey lines respectively.

Deletion of the SM22 homologue in yeast leads to increased actin dynamics and reduced levels of reactive oxygen species [13]. Here we describe SM22 depletion having an effect on actin organization by reducing prominent stress fibres to a more disorganized array (Figure 2) also suggestive of an increase in actin dynamics, with further evidence of an increase in actin dynamics revealed by the appearance of podosomes (Figure 5). In addition, invasive motility is also increased. A concomitant reduction in reactive oxygen species would potentially allow a cell depleted for SM22 to have an increased survival advantage in a hypoxic tumour environment. In order to test whether deleted SM22 levels would also lead to changes in cell's levels of reactive oxygen species, we subjected REF52 cells, either normal controls or SM22 depleted, to complete serum starvation for either 24 or 48 hours. Serum starvation is a recognized cellular stress that leads to elevated ROS in normal and tumour cells ([23,24] and references therein). As can be seen from Figure 6A, both 24 and 48 h serum starvation induced a greater than 15-fold increase in ROS levels in control REF52 cells. However, analysis of ROS levels in SM22 depleted REF52 cells (Figure 6B), revealed considerably reduced ROS levels at 24 h and 48 h (4-fold and 11-fold lower respectively than controls). Moreover untreated SM22-depleted cells had a baseline ROS level one third of wildtype cells. SM22 depletion *per se *appears to reduce ROS levels in REF52 cells, as was seen in yeast when the yeast orthologue Scp1 was deleted [13], but in addition SM22 depletion also appears to be protective against stressful stimuli that lead to ROS accumulation.

### Re-expression of SM22 in prostate tumour cell line PC3 reduces podosome formation

**Figure 7:**
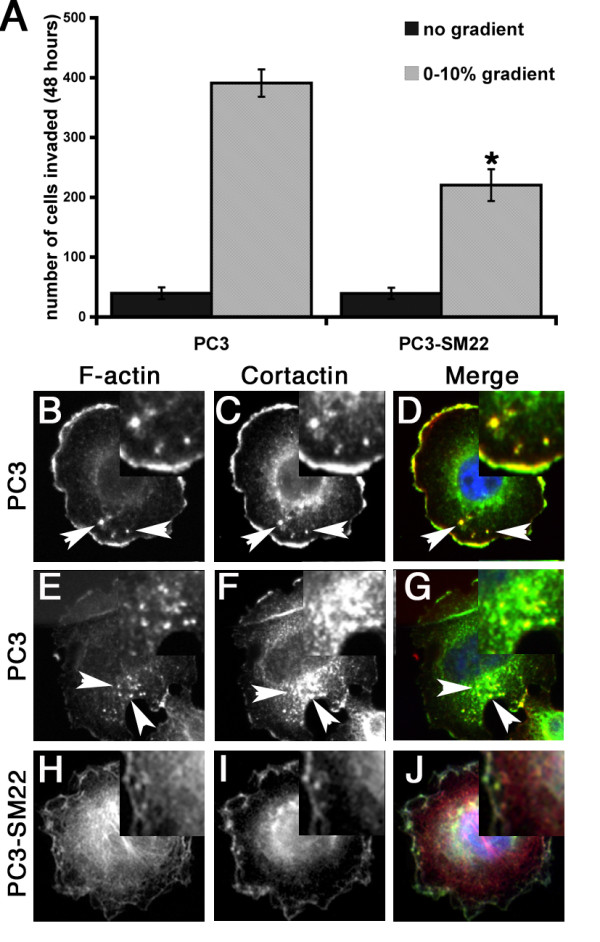
**SM22 re-expression reduces PC3 Matrigel invasion and podosome formation**. Re-expression of SM22 in PC3 cells reduces significantly the number of cells invading and traversing the filter in Matrigel coated Boyden chamber assays in response to a serum gradient (A); mean ± SEM 3 independent experiments, * p < 0.03 PC3 compared to PC3-SM22 in 0-10% gradient. PC3 cells stained for F-actin (B, E, H red in merge), cortactin (C, F, I green in merge). Cells in B-G are control PC3, cell in H-J is expressing SM22. Normal PC3 exhibit prominent ruffling membranes and podosomes (arrowed) as indicated by the co-staining of F-actin and cortactin, whereas SM22 expressing PC3 cells have reduced ruffling membranes and are devoid of podosomes.

Given that SM22 depletion in REF52 cells had a significant effect on actin morphology and increased Matrigel invasion, and that SM22 is also reduced in many tumour cells [5,25], we investigated the effect of manipulating SM22 levels in PC3 prostate cancer cells, a cell type that forms podosomes spontaneously [26]. As SM22 is not detectable by western blotting in PC3 cells (data not shown and see [27]), we therefore compared the ability of normal PC3, and PC3 overexpressing SM22 to form podosomes and to migrate through Matrigel coated Boyden chambers. As can be seen in Figure 7A, SM22 overexpression reduced the ability of PC3 cells to migrate through Matrigel in response to a serum gradient. Podosomes were detected using antibodies to a podosome component cortactin. PC3 cells can be seen to exhibit prominent ruffling membranes and podosomes (Figure 7B-G), as indicated by co-staining of F-actin and cortactin. However, SM22 expressing PC3 cells had reduced ruffling membranes and were devoid of podosomes (Figure 7H-J).

## Discussion

As we have demonstrated here, modulation in the levels of SM22 in fibroblast cells alters actin morphology which in turn affects their adhesion and migration phenotype. These changes are consistent with increases or decreases in the overall actin bundling activity in the cell resulting in decreased or increased actin dynamics respectively. Studies in yeast have revealed similar consequences for the effects of Scp1p, the yeast homologue of SM22, in actin dynamics and actin dependent processes such as endocytosis [11,12,28]. The actin binding and bundling activity of SM22 and related proteins is conferred via sequences c-terminal of the calponin homology (CH) domain including a short linker peptide and one to three calponin-like or 'CLIK^23^' repeats [29]. The presence of multiple CLIK repeats can increase the actin bundling properties of the protein thus stabilizing the actin cytoskeleton of the cell [29]. By the use of the *C. elegans *UNC-87 protein, which contains 7 CLIK repeats [30], Gimona and colleagues elegantly demonstrated that expressing increasing numbers of UNC-87 CLIK repeats progressively stabilized the actin cytoskeleton and inhibited the cell motility and growth in soft agar of breast cancer cell lines [29].

Stabilization of the actin cytoskeleton by the overexpression of an actin bundling protein might be expected to have an effect on properties of the cells that rely upon a dynamic actin cytoskeleton. However, the ability of CLIK repeat-containing proteins and for SM22 in particular to achieve this effect appears to be a more specific function that could be associated with suppression of the tumour phenotype. Numerous studies have identified SM22 as being downregulated in cancer [26,30-35] and some have even gone so far as to claim it as a tumour suppressor [5]. However, there are contradictory reports suggesting a positive correlation between an increase in SM22 levels and colon cancer metastasis to lymph node [31] and increased SM22 levels in gastric cancer [32], though in the latter study it was acknowledged that this was likely due to SM22 associated with the increased vascularisation of the tumour rather than in the tumour cells themselves. Proteomic profiling of prostate cancer cell lines revealed considerable variation in levels of SM22 in PC3 and LNCaP cells, even between different clonal variants of the same cell line [33]. Similar studies comparing prostate, colorectal and hepatocellular carcinoma also identified increased levels of SM22 associated with invasiveness [34].

Whilst the various studies investigating SM22 levels in different tumours appear to be at odds, one has to consider the cellular basis for the various cancers under scrutiny. SM22 is normally expressed in mesenchymal cells and not in epithelial cells, however in tumour cells undergoing an epithelial to mesenchymal transition (EMT), it may be that SM22 is re-expressed as a consequence of the adoption of the mesenchymal phenotype. A similar phenomenon occurs with other epithelial proteins in EMT, such as the E-cadherin to N-cadherin switch [35]. Therefore differences observed in the levels of SM22 in different tumours may be a reflection of several factors, including the tissue/cell of origin, whether it is a primary or secondary tumour, the cellular phenotype with respect to EMT, the precision of the sampling of the tumour if it was conducted on a tissue sample, and/or the relationship between a cell line and the original tumour if it is an *in vitro *study. Therefore SM22 may be upregulated in an adenocarcinoma during EMT, but downregulated in a sarcoma. Additionally SM22 appears to have a role in regulating transcription, with some genes being regulated in an SM22-dependent way [31,36] which could also modulate the tumour phenotype. In particular, relief of the repression of the matrix metalloproteinase MMP9 expression by SM22 in tumour cells where SM22 is downregulated [36] would lead to increased MMP9 levels. This could have a significant impact on cancer progression, especially invasion and metastasis. Whether the effect of SM22 re-expression suppressing the Matrigel invasion phenotype of PC3 cells is a consequence of reduced MMP9 expression or simply an effect on the dynamics of the actin cytoskeleton will require further analysis. This coupled with our observation that a reduction in SM22 levels also caused an increase in podosome and rosette-like structures in non-transformed REF52 cells might tend to argue in favour of SM22 acting as a tumour suppressor [5]. But the lack of a consistent association between a reduction in SM22 levels and tumour phenotype, and the contrary evidence of SM22 overexpression reducing growth in soft agar, [31] would tend to argue against SM22 being a true tumour suppressor. One additional explanation for why loss of SM22 might be advantageous in a tumour environment comes from its effect on the levels of reactive oxygen species (ROS) [13,37,38]. As we show here, SM22 depletion results in REF52 cells being less susceptible to stress-induced ROS production, in a manner analogous to the reduction in ROS levels seen upon Scp1P depletion in *S. cerevisiae*. Moreover, oxidative stress in diploid fibroblasts specifically upregulates SM22 expression via a TGF-β dependent mechanism, contributing to the senescent phenotype [38]. Reduction in SM22 levels would therefore tend to promote cell survival when cells are under stress, such as in a tumour environment, and may also contribute to increases in actin dynamics that favour metastatic potential.

## Conclusions

This study highlights some of the mechanisms for the apparent anti-oncogenic effect of SM22 at the cellular level. Others have found that the raised expression of this protein is associated with transforming and anti-transforming phenotypes but this is also known to be the case for many other actin cytoskeleton associated proteins, for example, gelsolin [39,40]. The present study is especially interesting since the down regulation of SM22 is associated with the appearance of podosomes, structures implicated in extracellular remodeling and invasion. These results open the way to understanding how these structures form and what the role of SM22 and other genes are in this important process.

## Methods

### Western Blotting

SDS-PAGE and western blotting were carried out as described previously [41]. Antisera against SM22α (ab10135, Abcam plc) NP22, NP25 [42], tubulin (Sigma) and actin (Sigma) were used for the detection of SM22 isoforms and for quantization of shRNA and RNAi knockdown.

### Cell Culture, Transfection, Immunofluorescence Microscopy and Generation of Stable Cell Lines

REF52 [43] and PT67 (Clontech) cells were cultured in Dulbecco's modified Eagle's medium (DMEM) and PC3 cells [44] in RPMI 1640, each supplemented with 10% foetal calf serum (FCS) (Invitrogen). All transfections were performed using Lipofectamine (Invitrogen) at 70% confluency with 2 μg of plasmid of interest per 35 mm dish. For immunofluorescence microscopy, cells were seeded on coverslips, and following treatment of transfection were fixed with 3.7% paraformaldehyde for 10 minutes and permeabilised with 0.05% Triton X-100 in PBS for 1 minute. Fixed cells were then incubated with Alexa 488-conjugation Phalloidin (Molecular Probes) to visualize F-actin, anti-SM22α antibody (ab10135, Abcam plc), cortactin (4F11, Upstate). Images were captured digitally by cooled CCD camera on a Leica DMIRE2 microscope. For the generation of stable cell lines, stable transfected cells were first selected with relevant antibiotic 48 hours post transfection. Stable clones were then selected.

### Cell Motility Assay

For cell migration assays, cells were allowed to adhere to 35 mm dishes overnight, then transferred onto a 37°C heated microscope stage within 5% CO_2 _chamber. Cell movements were captured on 2.5 minute time-lapse using a Leica DMIRBE microscope and Volocity software. The migratory paths of individual cells were tracked using ImageJ (version 1.38x) with Manual Tracking PlugIns. Movement and velocity from the initial point where calculated for each cell using Excel. 10 cells were analyzed for each triplicate independent experiment per cell line. Matrigel-coated Boyden chambers were purchased from BD Biosciences and cell invasion assays were performed and analyzed according to the manufacturers instructions, and using 10% serum in the lower chamber as chemoattractant.

### RNA interference

Potential 21mer sequences to be used for RNAi of SM22α were identified using the BBSRC Chick EST database RNAi target sequence prediction tool http://www.chick.umist.ac.uk/. The selected oligonucleotide sequences GCGTGATTCTGAGCAAGTTTTCAAGAGAAACTTGCTCAGAATCACGCTTTTTTACGCGT and ACGCGTAAAAAAGCGTGATTCTGAGCAAGTTTCTCTTGAAAACTTGCTCAGAATCACGC were cloned into pSIREN retroviral vector (Clontech). This vector was then transfected into the packaging PT67 cell line for production of retroviral SM22α siRNA vector. Target cells were infected twice with retrovirus before selection with puromycin. Sense control was previously generated in Ref52 cells using sense DG siRNA sequence [45]. Transient RNAi knockdown was achieved using pSiren-DNR-dsRed (Clontech).

### ROS Measurement

Control REF52, or SM22 depleted REF52 cells were grown at subconfluent levels in serum containing media as described above, or in media without serum for 24 and 48 hours. Following the indicated treatments, cells were incubated with 2',7'-dichlorodihydrofluorescein diacetate (H_2_DCFDA, Invitrogen, Karlsruhe, Germany) at 200 μg/μl for 1 hour at 37°C, washed in PBS, trypsinised and incubated with 100 μg/ml Propidium iodide (PI) for 15 minutes to visualize and exclude apoptotic/necrotic cells. Treated cells were then collected in PBS and analyzed using a Cyan Flow Cytometer (DakoCytomation) with Summit4.3 software.

## Authors' contributions

OT and JSM participated equally in the design and execution of the study and analysis. KRA and SJW conceived of the study, and participated in its design and coordination and helped to draft the manuscript. All authors read and approved the final manuscript.

## Supplementary Material

Additional file 1 **REF52 stably expressing siRNA constructs directed against SM22 show altered actin morphology**. Similar to results with REF52 cells transiently expressing SM22 RNAi (Figure 2 of main manuscript), REF52 cells with a stable depletion of SM22 levels also exhibited a qualitatively similar change in actin stress fibre organization (A) and as defined for Figure 2 of the main manuscript, with an overall reduction in stress fibre density and organization in cells lacking SM22, cf Figure 2. B, representative western blot of SM22α levels in siRNA control and depleted (KD) cells. C, quantization of stress fibre phenotypes in wildtype REF52 cells (black bars), siRNA control cells (grey bars) or SM22α depleted cells (white bars). Data are mean ± SEM or 3 independent experiments. Knockdown * p < 0.02 compared to wild type and p < 0.001 compared to siRNA control. No significant difference between knockdown and sense control (p > 0.05). Click here for file
