# Supplementary material for: Depletion of the actin bundling protein SM22/transgelin increases actin dynamics and enhances the tumourigenic phenotypes of cells
Source: BMC Cell Biol. 2012 Jan 18;13:1. doi: 10.1186/1471-2121-13-1 (PMC3280177; doi:10.1186/1471-2121-13-1)
Supplement: Additional file 1 — REF52 stably expressing siRNA constructs directed against SM22 show altered actin morphology. Similar to results with REF52 cells transiently expressing SM22 RNAi (Figure 2 of main manuscript), REF52 cells with a stable depletion of SM22 levels also exhibited a qualitatively similar change in actin stress fibre organization (A) and as defined for Figure 2 of the main manuscript, with an overall reduction in stress fibre density and organization in cells lacking SM22, cf Figure 2. B, representative western blot of SM22α levels in siRNA control and depleted (KD) cells. C, quantization of stress fibre phenotypes in wildtype REF52 cells (black bars), siRNA control cells (grey bars) or SM22α depleted cells (white bars). Data are mean ± SEM or 3 independent experiments. Knockdown * p < 0.02 compared to wild type and p < 0.001 compared to siRNA control. No significant difference between knockdown and sense control (p > 0.05). [file 1471-2121-13-1-S1.PDF]

Additional file 1

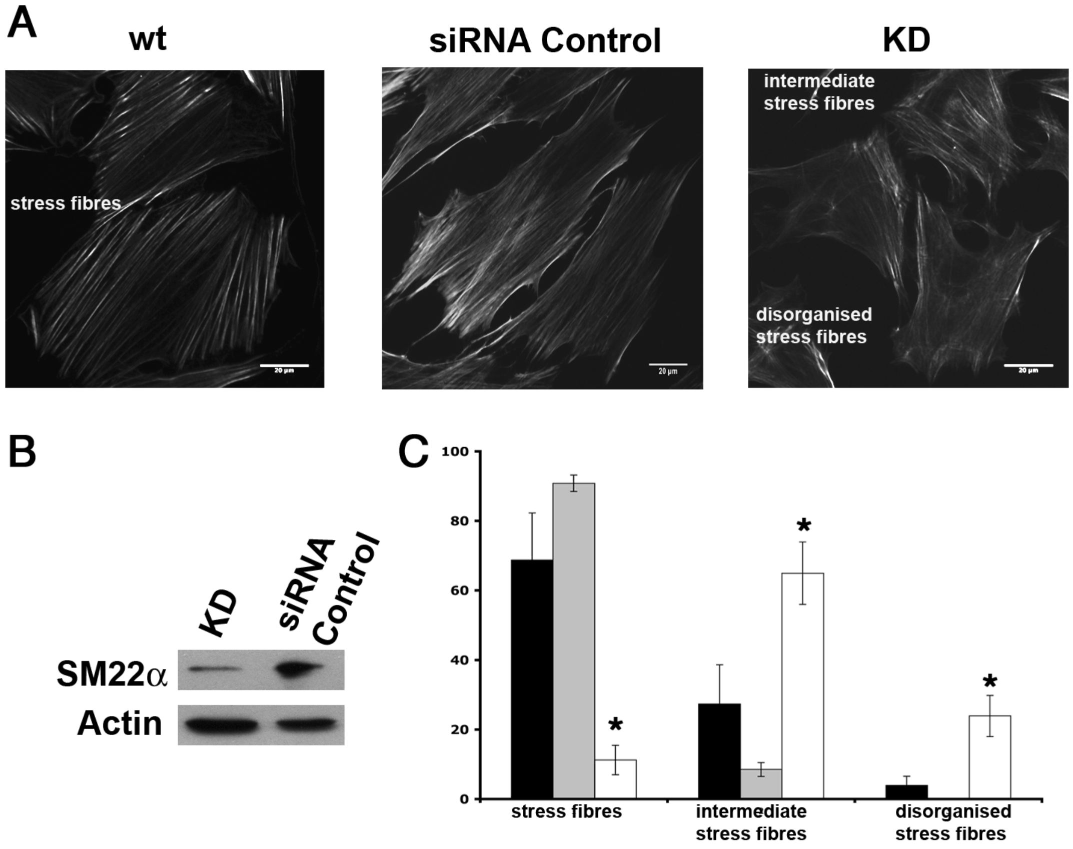

Figure S1. REF52 stably expressing siRNA constructs directed against SM22 show altered actin morphology. Similar to results with REF52 cells transiently expressing SM22 RNAi (Figure 2 of main manuscript), REF52 cells with a stable depletion of SM22 levels also exhibited a qualitatively similar change in actin stress fibre organization (A) and as defined for Figure 2 of the main manuscript, with an overall reduction in stress fibre density and organization in cells lacking SM22, cf Figure 2. B, representative western blot of SM22α levels in siRNA control and depleted (KD) cells. C, quantization of stress fibre phenotypes in wildtype REF52 cells (black bars), siRNA control cells (grey bars) or SM22α depleted cells (white bars). Data are mean±SEM or 3 independent experiments. Knockdown * $p<0.02$ compared to wild type and $p<0.001$ compared to siRNA control. No significant difference between knockdown and sense control ($p>0.05$).
